# Effects of carbohydrate restriction on heart rate variability in people with type 2 diabetes during weight loss. A secondary analysis of a randomized controlled trial

**DOI:** 10.1016/j.ajpc.2026.101544

**Published:** 2026-03-10

**Authors:** Philip Weber, Mads N. Thomsen, Mads J. Skytte, Amirsalar Samkani, Jens J. Holst, Ahmad Sajadieh, Preman Kumarathurai, Sten Madsbad, Faidon Magkos, Thure Krarup, Steen B. Haugaard

**Affiliations:** a Department of Endocrinology, Copenhagen University Hospital – Bispebjerg and Frederiksberg, Copenhagen, Denmark; b Department of Clinical Physiology and Nuclear Medicine, Copenhagen University Hospital – Bispebjerg and Frederiksberg, Copenhagen, Denmark; c Novo Nordisk Foundation Centre for Basic Metabolic Research and Department of Biomedical Research, University of Copenhagen, Copenhagen, Denmark; d Department of Cardiology, Copenhagen University Hospital – Bispebjerg and Frederiksberg, Copenhagen, Denmark; e Department of Clinical Medicine, University of Copenhagen, Copenhagen, Denmark; f Department of Cardiology, Odense University Hospital, Odense, Denmark; g Department of Endocrinology, Copenhagen University Hospital – Amager and Hvidovre, Hvidovre, Denmark; h Department of Nutrition, Exercise and Sports, University of Copenhagen, Copenhagen, Denmark

## 1 Introduction

Low heart rate variability (HRV) is associated with impaired glycemic control [1] and represents a key feature of cardiac autonomic neuropathy (CAN) [2]. In individuals with type 2 diabetes (T2D), 24-hour HRV assessment is proposed to be a sensitive measure for CAN and has been associated with increased risk of atherosclerosis [3]. In T2D, low HRV independently correlates with atherosclerotic cardiovascular events [4].

We showed previously that modest weight loss (6 % from baseline) induced by a carbohydrate-restricted diet improved glycated hemoglobin (HbA_1c_) and triacylglycerol (TAG) levels in the blood and liver to a significantly greater extent than the same weight loss induced by a high-carbohydrate diet [5]. We hypothesized that these additional metabolic benefits of carbohydrate restriction could lead to improved autonomic nerve function of the heart, reflected by increased HRV and lower resting heart rate.

## 2 Methods

### 2.1 Study population, design and intervention

**Fig. 1:**
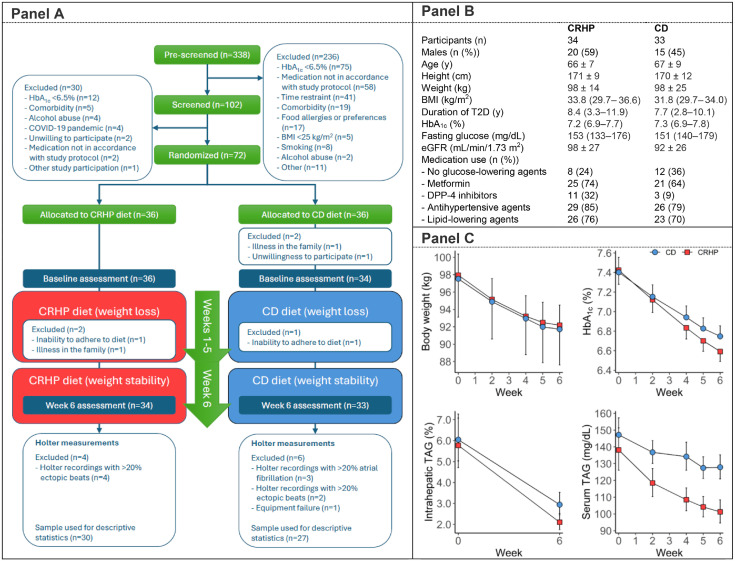
(Panel A) Participant flow diagram. (Panel B) Baseline characteristics of the study population from the main trial (5). Data are presented as mean ± SD or median (25th–75th percentile); number of participants, males, and medication use are presented as counts and percentages. (Panel C) Effects on body weight and HbA_1c_ (arithmetic mean ± SEM) and intrahepatic and serum TAG concentrations (geometric mean ± SEM), representing the primary and secondary outcomes of the main study previously reported in (5). Abbreviations: CD, conventional diabetes diet; CRHP, carbohydrate-reduced high-protein diet; eGFR, estimated glomerular filtration rate; DPP-4, dipeptidyl peptidase-4; T2D, type 2 diabetes; TAG, triacylglycerol.

The study design has been described in detail previously, along with the primary and secondary outcomes [5]. This was an open-label, parallel, randomized controlled trial designed to investigate the effects of dietary carbohydrate restriction on glycemic control and intrahepatic TAG content in people with T2D (Fig. 1, Panel A). Adults diagnosed with T2D, HbA_1c_ between 6.5–11 %, BMI >25 kg/m^2^, and eGFR >30 mL/min/1.73 m², receiving glucose-lowering therapy restricted to metformin and dipeptidyl peptidase-4 (DPP-4) inhibitors, were included. Exclusion criteria have been elaborated previously [5].

Participants were randomly assigned to 6 weeks of a carbohydrate-reduced high-protein (CRHP) or a conventional diabetes (CD) diet, containing 30/50 energy-percent (E %) from carbohydrate, 30/17E % from protein, and 40/33E % from fat, in a 1:1 ratio in blocks of random size (4–12 participants) by a third-party using R (R Core Team, 2023). The dietary intervention aimed for a 6 % reduction in baseline body weight within the first 5 weeks, followed by 1 week of isocaloric feeding (containing the same relative macronutrient composition) to avoid the acute effects of negative energy balance on end-of-study testing. The diets, including 3 main meals and optional snacks daily, were prepared and packaged at the metabolic kitchen of the Department of Nutrition, Exercise and Sports, University of Copenhagen, and supplied to participants regularly and free of charge. Participants were required to consume only the food provided and avoid all calorie-containing beverages, including alcohol. They were also instructed to maintain their habitual level of physical activity throughout the study.

### 2.2 Holter monitoring

Participants wore a 3-lead Holter monitor system (Faros™, Bittium, Oulu, Finland) for 48 h at baseline and during the last week of the intervention (week 6). The last 24 h of each recording were selected and used to estimate HRV metrics. Before analysis, Holter recordings were edited by an investigator blinded to dietary treatment using relevant software (Cardiac Navigator™, Bittium, Oulu, Finland). Only recordings that had a regular sinus rhythm, more than 80 % of the total recording, were included for data analysis. To evaluate overall variability across all cyclic components responsible for cardiac variability, we investigated the 24-hour standard deviation of all normal-to-normal (NN) interbeat intervals (SDNN). We evaluated the average NN of each consecutive 5-minute segment (SDANN) to investigate long-cycle variability (e.g., circadian rhythm, sleep-wake transition, behavioral/activity-related modulation), and the square root of the mean difference of successive NN intervals (RMSSD) to evaluate short-cycle variability. High- and low-frequency (HF: 0.15–0.40 Hz, reflecting parasympathetic activity; LF: 0.04–0.15 Hz, reflecting a mixture of sympathetic and parasympathetic incl baroreflex activity) power were measured. LF/HF ratio was derived and interpreted to reflect the relative balance between sympathetic and parasympathetic autonomic influences. Frequency-domain measures were computed using a Fast Fourier Transform (FFT) approach, applying Welch’s method for spectral density estimation [6].

### 2.3 Statistical analysis

The sample size calculation was based on the primary and secondary outcomes (HbA_1c_ and intrahepatic TAG content), which have been described in full previously [5]. In short, we calculated a requirement of 80 participants to achieve sufficient statistical power, with an expected 20 % attrition rate.

Continuous variables were analyzed using constrained linear mixed models (CLMMs), which inherently adjust for baseline differences, assuming an unstructured covariance pattern to account for repeated measurements. All HRV analyses were adjusted for the covariates: sex, age, BMI, diabetes duration and HR and log-transformed, if necessary, based on model residuals. Only participants with both HRV measurements (baseline and week 6) of high quality (defined as >80 % sinus rhythm) were included in the descriptive statistics. All available data were included in the CLMMs, which can handle missing values. Statistical significance was set at *p* < 0.05.

## 3 Results

We enrolled 72 people with T2D of of whom 67 completed the 6-week follow-up (Fig. 1, Panel A), thereby providing sufficient power [5]. Baseline characteristics were similar between groups (Fig. 1, Panel B), except for a higher proportion of males and individuals on DPP-4 inhibitors in the CRHP group compared with the CD group. Both groups achieved a 5.8 kg weight loss, equivalent to 5.9 % of baseline body weight. As reported previously [5], HbA_1c_ decreased in both diet groups, with a 25 % greater reduction observed in the CRHP group compared with the CD group, accompanied by greater reductions in TAG in the liver (26 %) and in blood (18 %) (Fig. 1, Panel C).

### 3.1 HRV and HR

Holter recordings showed sinus rhythm median (25th-75th percentile) 99.5 (95.2–99.8) % of the time at baseline and 99.0 (96.0–99.8) % at week 6. A total of 10 participants were excluded from the descriptive statistics due to equipment malfunction (CD 1) or <80 % sinus rhythm of at least one recording (CRHP 4, CD 5), leading to 57 participants being included in the descriptive statistics (Fig. 1, Panel A). The reasons for recordings of <80 % sinus rhythm were episodes of known paroxysmal atrial fibrillation (*n* = 2), persistent atrial fibrillation (*n* = 1), excess ectopic beats (*n* = 6). The ten participants excluded did not differ from the 57 participants providing HRV data on baseline or week 6 (data not shown).

In both diet groups, we observed significant improvements in 24-hour HR and all measures of HRV (except for RMSSD in the CRHP group and LF/HF ratio) during the 6 weeks, but without any difference between the groups (Table 1).

**Table 1:** Heart rate and HRV measures before and after matched weight loss by a CD or a CRHP diet in individuals with overweight or obesity and type 2 diabetes.

Variable	CRHP diet			CD diet			Between diets	
	Baseline	Change	*n*	Baseline	Change	*n*	Difference	*p*-value
Heart rate (bpm)	76 (73, 79)	−6 (−9, −4)	30	72 (68, 76)	−8 (−10, −6)	27	2 (−1, 5)	0.1
Time-domain HRV								
SDNN (ms)	116 (102, 130)	34 (18, 50)	30	124 (111, 138)	36 (23, 49)	27	0 (−17, 17)	1.0
SDANN (ms)	106 (91, 121)	30 (14, 46)	30	111 (97, 124)	31 (18, 44)	27	1 (−16, 18)	0.9
RMSSD (ms)	26 (22, 30)	2 (−1, 5)	30	29 (24, 33)	5 (1, 9)	27	−3 (−7, 2)	0.3
Frequency-domain HRV								
HF (ms^2^)^a^	124 (91, 169)	30 (2, 64)	30	175 (127; 241)	37 (9, 72)	27	−4 (−26, 27)	0.7
LF (ms^2^)^a^	325 (244, 432)	39 (14, 69)	30	404 (297, 548)	43 (22, 70)	27	−1 (−22, 17)	1.0
LF/HF	3.2 (2.4, 4.0)	0.1 (−0.4, 0.6)	30	2.6 (2.0, 3.3)	0.2 (−0.3, 0.6)	27	0.0 (−0.6, 0.6)	1.0

CD, conventional diabetes; CRHP, carbohydrate-reduced high-protein; HF, high-frequency; HRV, heart rate variability; LF, low-frequency; RMSSD, root mean square of successive differences; SDANN, standard deviation of the average normal-to-normal interbeat interval (5-minute segments); SDNN, standard deviation of normal-normal interbeat interval. Values are expressed as arithmetic or geometric means (95 % confidence intervals) according to whether or not data were log-transformed to meet the assumption of normality distribution. Between-diet differences are estimated marginal means (CRHP vs CD) derived from a constrained linear mixed model, using all available data, adjusted for baseline values, sex, age, diabetes duration, and heart rate.

a Data are presented as geometric mean (95 % confidence interval) and relative change (%) from baseline.

## 4 Discussion

In this study, modest weight loss (5.9 kg) over 6 weeks improved HRV parameters in people with T2D and obesity, regardless of the macronutrient composition of the diet.

HRV improvements in this study may be mediated by several mechanisms driven by weight loss, such as reduced systemic low-grade inflammation, lower oxidative stress, and lower metabolic stress on the sinoatrial node pacemaker cells, which are key regulators of HRV [3]. The similar weight loss achieved between groups in this study likely explains the comparable improvements; thus, our results align with previous studies showing that weight loss is a primary driver of improved HRV in individuals with T2D [7,8]. In people with newly diagnosed T2D, the pathophysiological driver of CAN is suggested to be tied to parasympathetic impairment driven by insulin resistance rather than hyperglycemia per se [9]. This could explain why the improvements in HRV observed in this study appear to be driven more by weight loss and reductions in visceral adiposity than by changes in glucose levels.

Although the present study lack a weight-stable control group to formally estimate the independent effect of weight loss on HRV measures, an increase in 24-hour SDNN of 30 % in the present study after diet-induced weight loss contrasts the 25 % reduction observed in people with ischemic heart disease and T2D undergoing glucagon-like-peptide 1 receptor agonist therapy (liraglutide) for 12 weeks and loosing body weight [10]. The strengths of our study include its randomized controlled design, full diet provision, and high-quality, long-term HRV data acquisition in a relatively large population.

## 5 Conclusion

In individuals with T2D and obesity, greater improvements in glucose and lipid metabolism after modest weight loss induced by replacing dietary carbohydrate with protein and fat did not translate into additional improvements in HRV measures.

## Ethics approval and consent to participate

The study protocol adhered to the Declaration of Helsinki and was approved by the Health Ethics Committee of Copenhagen and the Danish Data Protection Agency.

## Funding

Grants from The Danish Dairy Research Foundation, Arla Foods amba, The Novo Nordisk Foundation, and Copenhagen University Hospital – Bispebjerg and Frederiksberg supported this study. The funders had no role in the study’s design, execution, data interpretation, or publication decisions.

## CRediT authorship contribution statement

**Philip Weber:** Writing – original draft, Visualization, Validation, Software, Investigation, Formal analysis, Data curation. **Mads N. Thomsen:** Writing – review & editing, Validation, Supervision, Project administration, Investigation. **Mads J. Skytte:** Writing – review & editing, Conceptualization. **Amirsalar Samkani:** Writing – review & editing, Conceptualization. **Jens J. Holst:** Writing – review & editing, Conceptualization. **Ahmad Sajadieh:** Writing – review & editing, Conceptualization. **Preman Kumarathurai:** Writing – review & editing, Conceptualization. **Sten Madsbad:** Writing – review & editing, Conceptualization. **Faidon Magkos:** Writing – review & editing, Conceptualization. **Thure Krarup:** Writing – review & editing, Supervision, Resources, Funding acquisition, Conceptualization. **Steen B. Haugaard:** Writing – review & editing, Supervision, Resources, Funding acquisition, Conceptualization.

## Declaration of competing interest

The authors declare the following financial interests/personal relationships which may be considered as potential competing interests: none.
